# A potential effect of psilocybin on anxiety in neurotic personality structures in adolescents

**DOI:** 10.3325/cmj.2021.62.528

**Published:** 2021-10

**Authors:** Marija Bogadi, Snježana Kaštelan

**Affiliations:** 1 Psychiatric Hospital for Children and Adolescents, Zagreb, Croatia *marija.bogadi@gmail.com*; 2 University of Zagreb School of Medicine, Zagreb, Croatia

Psilocybin is a naturally occurring psychedelic substance present in a variety of Psilocybe mushroom species (1). It has been used in different cultures since prehistoric times to induce hallucinations and altered states of consciousness (2). Upon ingestion, psilocybin is transformed into a pharmacologically active ingredient psilocin. It affects a number of serotonin receptors, but has the highest affinity for serotonin 2A receptor. The effects of psilocybin are dose-dependent and include perceptual, cognitive, and emotional changes (1,3-5). The drug improves mood instantly, but the effects may last over several months, during which time psychotherapeutic interventions can be used (5-7). In 1959, while working for Sandoz Pharmaceuticals, Albert Hofmann isolated purified psilocybin from Psilocybe mushrooms, which was registered under the commercial name Indocybin (8). However, increased misuse of psychedelic drugs in the late 1960s led to the prohibition of production, trade, and consumption of hallucinogenic drugs (9). Even though psilocybin was banned in many countries, including Croatia, over time its therapeutic benefit has been demonstrated. Several studies have shown its usefulness in managing treatment-resistant depression, anxiety, and depression in life-threatening diseases, obsessive-compulsive disorder, alcohol and smoking dependence, posttraumatic stress disorder, and cluster headaches (10-16). However, the available research has not covered its impact in children and adolescents.

Social phobia is marked by a persistent fear of embarrassment or humiliation in social situations. Anticipatory anxiety and avoidance occur when an individual is under scrutiny during public speaking or other public activities. This highly prevalent and chronic disorder significantly impairs psychosocial functioning, with pharmacological and psychosocial treatments showing limited results (17,18). However, psychedelic-assisted therapy used in combination with psilocybin may offer an additional treatment option for patients who fail to respond to conventional treatments (18). Psilocybin is generally well-tolerated, with the most common side effects being transient anxiety, mild increases in heart rate and blood pressure, nausea, headaches, blurred vision, dizziness, weakness, and tremor (1,16,19-21).

This article reports on a 16-year-old male patient treated in a psychiatric clinic with the symptoms of social distancing, increased intensity of anxiety, and poor school performance. He experienced learning disabilities, lack of motivation for schoolwork with poor academic results, and isolation from his peers and teachers. The transfer to a new school failed to bring about any improvement. The adolescent started attending a psychotherapeutic group but found it difficult to communicate with other members and express his feelings, so he often avoided sessions due to anxiety. He occasionally consumed marijuana, which failed to reduce anxiety and improve his functioning. After being offered psilocybin by a friend, he consumed two grams of Psilocybe mushrooms, corresponding 20 to 30 mg of the substance, on three separate occasions over an 18-month period. The patient described the effect as a feeling of sublimity and enlightenment. After this experience, he more frequently participated in group activities, communicated better with other group members, expressed emotions without inhibition, and felt less anxious. Such rapid and evident improvement of group psychotherapy results has not yet been reported. In fact, it has not been shown that psychotherapy alone could lead to such sudden and long-term changes in patients’ mental status.

The patient's feelings of “connection with people around him” and “sublimity” can be explained by the activation of certain neural systems of the brain, particularly the so-called seeking systems, which play a role in fighting depression and anhedonia. In other words, people are motivated to accept pleasure and avoid discomfort. These neuroanatomical changes form the basis for opioid addiction. Once this system is activated, the experience of “solving the problem of anhedonia” remains in the memory engrams and is quickly and easily accessible. Psilocybin increases the concentration of extracellular serotonin and dopamine in the nucleus accumbens and the median prefrontal cortex. This system, particularly nucleus accumbens, is an important part of the “seeking system.” In addition, the medial bundle of the forebrain, which is connected to the nucleus accumbens and is located in the immediate vicinity of the median prefrontal cortex, could be simultaneously activated. Accordingly, even if these neural areas are stimulated only once or twice, in the patient’s memory an impression is created that “everything will be fine.” The guilt that the patient experiences helps him to refrain from taking this substance again. However, he is aware that by consuming psilocybin his problem was “magically solved.” The feeling that “everything can be fine” continues to exist only at the level of an idea due to the stimulation of the mentioned neural circuits and nucleus. The same system is also stimulated by frequent psychotherapeutic treatment. During psychotherapeutic treatment, a sense of satisfaction arises from repeated conflict resolution or pleasure due to identifying with the psychotherapist. In addition, separation and reuniting subconsciously suggest that although the patient is alone, he or she will be reconnected with his favorite object. In this way, a so-called safe attachment is established, which creates new collaterals and dendritic shoots in the mentioned neural pathways. Ideally, completed psychotherapy enhances “collateral and dendritic” neural pathways that increase patient’s confidence in other people, enabling him or her to function in everyday life (6,7,22-27).

Conventional therapy often shows limited effectiveness in resolving the symptoms of anxiety and depression (5,9,16,18). The present case highlights the potential benefit of psilocybin when used concomitantly with psychotherapy led by an experienced therapist in adolescents with neurotic personality structures and generalized anxiety disorder and social phobia. To date, only a few double-blind studies have investigated the use of psilocybin in psychiatric treatment, with a sample size of fewer than 200 (9). Further research is warranted to determine the value of psychedelic substances in psychiatry. Based on the studies to date, the European Medicine Agency and Food and Drug Administration have approved a multicenter multinational trial of psilocybin that began in early 2019, with the first results expected during 2021 (9).

Although psilocybin use entails some level of risk and abuse potential, there is no strong evidence of physical or psychological dependence, and it can be considered safe under medical supervision (1,2,6,22). Nevertheless, it should be studied in combination with psychotherapy, rather than as a stand-alone drug treatment. Psychological support is essential when applying psilocybin or other psychedelics in practice or clinical trials (20,28). Likewise, psilocybin may promote long-lasting positive changes even upon single use (5,6). Drug-assisted psychotherapy may offer another option for anxiety and depression treatment in addition to the pharmacological and psychological treatments currently available in psychiatry. Further research needs to evaluate the potential of psilocybin in the management of anxiety and other psychiatric disorders.
